# Purification and Characterization of an Active Principle, Lawsone, Responsible for the Plasmid Curing Activity of *Plumbago zeylanica* Root Extracts

**DOI:** 10.3389/fmicb.2018.02618

**Published:** 2018-11-08

**Authors:** Rajashree Bhalchandra Patwardhan, Prashant Kamalakar Dhakephalkar, Balu Ananda Chopade, Dilip D. Dhavale, Ramesh R. Bhonde

**Affiliations:** ^1^Department of Microbiology, Haribhai V. Desai College of Arts, Science and Commerce, Pune University, Pune, India; ^2^Bioenergy Group, Agharkar Research Institute, Pune, India; ^3^Department of Microbiology, Savitribai Phule Pune University, Aurangabad, India; ^4^Department of Chemistry, Savitribai Phule Pune University, Pune, India; ^5^School of Regenerative Medicine – Manipal Academy of Higher Education, Bengaluru, India

**Keywords:** lawsone(2-hydroxy-1, 4 naphthoquinone), *Plumbago zeylanica*, extraction, purification, plasmid curing, antibiotic resistance

## Abstract

Plasmid curing is the process of obviating the plasmid encoded functions such as antibiotic resistance, virulence, degradation of aromatic compounds, etc. in bacteria. Several plasmid curing agents have been reported in literature, however, no plasmid curing agent can eliminate all plasmids from different hosts. Hence, there is always a need for novel plasmid curing agents that can be effectively used for reversal of plasmid encoded functions such as virulence, antibiotic resistance, etc. In the present study, an active principle responsible for the plasmid curing activity was purified from roots of *Plumbago zeylanica* by bioassay guided fractionation and identified as 2-hydroxy-1,4-naphthoquinone (lawsone), on the basis of spectral and analytical data such as NMR, GCMS, FTIR. Plasmid curing activity of lawsone was observed against reference as well as wild plasmids (pBR322, pRK2013, R136, pUPI281, and pUPI282) residing in a range of hosts. Curing of plasmid was confirmed by agarose gel electrophoresis. MICs of antibiotics against *A. baumannii* A24 (pUPI281) and *E. coli* (pRK2013) decreased significantly in presence of lawsone suggesting synergy between lawsone and antibiotics. Lawsone also inhibited transfer of plasmid pRK2013 to *E. coli* either by transformation or conjugation. Viability assays (MTT) revealed that lawsone was not toxic to mammalian cells. Thus, the present investigation has revealed lawsone as an effective plasmid curing agent capable of suppressing development and spread of antibiotic resistance. Further, lawsone has important application in basic research to identify phenotypes encoded by the plasmids in plasmid curing experiments. To the best of our knowledge this is the first report of plasmid curing activity of lawsone isolated from roots of *P. zeylanica.*

## Introduction

Plasmids are independent, circular, self-replicating extra-chromosomal DNA elements with characteristic copy numbers within the host. Various properties encoded by plasmid include resistance to antibiotics and heavy metals, degradation of hydrocarbons, synthesis of bacteriocins and antibiotics, etc. Plasmid mediated antibiotic resistance can be transferred easily from one bacterium to another by transformation, conjugation or mobilization (Opal et al., 2000). Plasmid encoded resistance to multiple antibiotics has been increasingly recognized as a major challenge in the treatment of infections. In addition to the antibiotic resistance, some bacterial plasmids confer pathogenicity as well, to the host cell (Saunders, 1981).

Plasmid curing agents are the chemicals or physical agents that inhibit the replication of plasmid resulting in subsequent elimination of such plasmids from the host population after several replication cycles. Obtaining plasmid cured derivatives is desired in the investigation of bacterial harboring plasmids. Comparison of plasmid harboring strain and its cured derivative allows assigning phenotypic characters to genes located on plasmids. Simultaneous loss of a particular character by curing, gives a strong indication of its plasmid borne genetic character (Trevors, 1986). Further, plasmid curing converts the antibiotic resistant bacterial cells into sensitive ones (Molnar, 1988). Thus, elimination of R-plasmids makes the antibiotic therapy effective. Novel strategies to struggle antimicrobial multidrug resistance are required, and plasmid curing, and anti-plasmid strategies could reduce antimicrobial resistance genes frequency and sensitize bacteria to antibiotics (Michelle et al., 2018).

Plasmid curing agents reported in the scientific literature include sodium lauryl sulfate, ethidium bromide, acridine orange, etc. (Bouanchaud et al., 1969; Chopade et al., 1994). However, it is known from earlier studies that acridine dyes and ethidium bromide cannot be used *in vivo* because of their mutagenicity, carcinogenicity, teratogenicity while SDS, because of its detergent action (Shriram et al., 2010). Hence, these curing agents are not useful in controlling the spread of antibiotic resistance in hospital environment. The curing agents have been effectively used to study plasmid encoded phenotypes in various laboratory studies. Till date no curing agent is known that can universally cure all plasmids from bacterial population. So non-toxic and highly effective plasmid eliminating agents are required to be developed constantly.

Plants are known to produce diverse bioactive substances of chemotherapeutic value (Chopra et al., 1992; Samy and Ignacimuthu, 2000). Ahmad et al. (2000) reported ability of aqueous extracts of *Plumbago zeylanica* to eliminate plasmid encoded antibiotic resistance in *Escherichia coli*. However, active principle responsible was not purified and identified.

In the view of this background, root extracts of *P. zeylanica* were explored for the presence of novel plasmid curing agent that was effective against antibiotic resistant plasmids in *Escherichia coli, Salmonella* Typhi as well as *Acinetobacter baumannii*. In the present investigation, lawsone was purified from the roots of *P. zeylanica* and identified as a plasmid curing agent capable of reversing multiple antibiotic resistance in broad range of clinical isolates as well as reference strains.

## Materials and Methods

### Plant Material

*P. zeylanica* plants were collected from Western Ghat region of India. The roots were harvested, dried in shade, powdered and used for further extraction procedures. The plant material was authenticated by Botanical Survey of India, Ministry of Environments and forests, Government of India. A voucher specimen (RBPUP1) is deposited at the Herbarium of Botanical Survey of India, Office of Joint Director, Pune, India.

### Extraction and Purification of Plasmid Curing Agent From *P. zeylanica* Roots

Active principle responsible for the plasmid curing activity was purified by the bioassay guided fractionation procedure as described previously (Shriram et al., 2008). Air-dried and powdered roots (2.5 kg) of *Plumbago zeylanica* were successively extracted in Soxhlet apparatus with petroleum ether, cyclohexane, benzene, diethyl ether, chloroform, acetone, ethanol, and methanol (according to eluotropic series based on polarity) at boiling temperature for respective solvents (Shriram et al., 2008). Each extract was filtered and concentrated to dryness under vacuum on a rotary evaporator (Heidolph-Germany) and dissolved in 10 ml of dimethyl sulfoxide (DMSO). Isolation and purification of active compounds from *P. zeylanica* roots was performed by column chromatography with silica gel (100–200 mesh) (Sadasivam and Manickam, 1992). Ethanol extract showing antimicrobial and plasmid curing activity was then coarsely fractionated over silica gel using a stepwise gradient solvent system consisting of hexane: ethyl acetate (9:1, 8:2, 7:3, 6:4, 5:5, 4:5, 3:6) to separate the respective fractions (25–40 μm, 3′ 50 cm, eluent hexane-ethyl acetate, flow rate 3 ml/min). TLC analysis was carried out on 0.25 mm precoated silica gel sheets (polygram sil Gluv 254). Chromatographic fractions as well as pure compounds were monitored by TLC, detected by UV light at 250 nm (UV GL-25 Mineralight lamp) and color reaction by spraying with a solution of 2% 2,4-dinitro phenyl hydrazine in methanolic sulphuric acid followed by 5 min heating at 100°C. A total of 83 sub-fractions (10 ml) were collected and monitored by TLC. Sub-fractions 17–23 yielded (64 mg), a single pure compound detected by TLC (hexane-ethyl acetate 7:3, *R*_f_ = 0.617). This compound was further purified by preparative TLC (Macherey-Nagel-Germany Precoated TLC plates SIL G-200 UV_254_ 2 mm thickness) using hexane-ethyl acetate solvent system.

### Characterization of Purified Plasmid Curing Agent

Melting point was determined in degree Celsius (°C) with Thomas Hoover Capillary Melting point apparatus (New Jersey, United States). IR spectra were recorded with Perkin Elmer 1600 FTIR and Shimadzu FTIR spectrophotometer as a thin film or in nujol mull or using KBr pellets and were expressed in cm^-1^. Spectral data on GCMS was acquired with the direct insertion probe on a Shimadzu spectrometer at 70 eV. Isolated compound was analyzed for structure elucidation by techniques such as ^1^H nuclear magnetic resonance (NMR) and ^13^C NMR (Choudhary and Atta-ur-Rahman, 1997). The ^1^H NMR spectra (300 MHz) were recorded in CDCl_3_ as a solvent on a Varian (mercury) instrument. ^1^H NMR chemical shifts were expressed in δ (ppm) units, downfield to internal standard TMS (Daniel, 1991). Assignment of signals was confirmed by decoupling experiments.

### Microbial Strains and Culture Conditions Used

Bacterial isolates and standard plasmids used in this study are enlisted in Tables 1, 2. Clinical isolates of *Acinetobacter* were identified based on their morphological, cultural and biochemical characteristics according to the Bergey’s Manual of Systematic Bacteriology (Kloos and Schleifer, 1986) and using API20NE system (Biomeraux, France) as per manufacturer’s instructions. All strains were preserved as glycerol stocks at -20°C or as agar slants at 4–8°C. All bacterial cultures were grown on Luria agar at 37°C (Hi Media, Mumbai, India).

**Table 1:** Antibacterial activity of lawsone from *P. zeylanica* ethanol root extract.

Sr. No.	Bacterial strains	Strain designation	MIC (μg/disk)
1	*P. aeruginosa*	PUPU103	800
2	*S. aureus*	PUPU107	400
3	*S.* Typhi	PUST112	200
4	*B. cereus*	PUBL123	200
5	*E. coli*	PUUR119	800
6	*S. dysenteriae*	PUST115	400
7	*B. subtilis*	PUBL126	200
8	*S. marcescens*	PUUR120	>1600
9	*P. mirabilis*	PUUR121	>1600
10	*K. pneumoniae*	PUSP134	800
11	*Enterobacter*	PUST114	800
12	*A. baumannii*	PUBL130	800

*MIC (minimum inhibitory concentration) was detected by disk diffusion assay using 10^*5*^ cells as inoculum on NA for bacteria. DMSO without lawsone, used as a solvent control, did not inhibit growth of any of the bacterial cultures tested. All the strains were isolated from clinical specimens like pus, stool, blood, urine, and sputum*.

**Table 2:** Plasmid curing with purified lawsone.

Strain and plasmid	Size of plasmid	Antibiotic resistance^@^	MIC (μg/ml)	SIC (μg/ml)	Curing efficiency (%)
*E. coli* K12 (RP4) MTCC 391	56.4 kb	Ap^r^, Km^r^, Tc^r^	512	256	ND
*E. coli* (pBR 322) MTCC 78	4.3 kb	Ap^r^, Tc^r^	800	512	11 ± 1.4
*E. coli* (pRK 2013) MTCC 398	48.0 kb	Ap^r^, Km^r^	800	512	20 ± 1.4
*P. aeruginosa* (RIP64) MTCC 1262	95 Mdal	Gm^r^, Cm^r^	512	256	ND
*S.* Typhi (R136) MTCC 1264	59 kb	Tc^r^	800	512	4.2 ± 0.3
*B. subtilis* (pUB110) MTCC 1558	4.5 kb	Km^r^, Nm^r^	800	512	ND
*A. baumannii* A23 (pUPI280) (present study)	__	Ap^r^, Gm^r^, Km^r^, Cm^r^, Am^r^	1024	800	ND
*A. baumannii* A24 (pUPI281) (present study)	__	St^r^, Ap^r^, Gm^r^, Ak^r^	800	512	13.5 ± 1.4
*A. baumannii* A26 (pUPI282) (present study)	__	St^r^, Ap^r^, Gm^r^, Ak^r^, Lf ^r^	512	256	8.5 ± 0.7

*MIC, minimum inhibitory concentration; SIC, sub inhibitory concentration. ND, none of the 300 colonies tested showed curing of plasmid. ^@^Am, amoxicillin; Ak, amikacin; Ap, ampicillin; Cm, chloramphenicol; Gm, gentamicin; Km, kanamycin; Lf, lomefloxacin; Nm, neomycin; St, streptomycin; Tc, tetracycline; **MTCC (Reference cultures),** Microbial Type Culture Collection, Chandigarh, India*.

### Determination of Resistance to Antibiotics

Antibiotic resistance profile was determined by the disk diffusion method (Bauer et al., 1966). Multi-disks containing antibiotics (Don Whitley Scientific Equipments, Mumbai, India) were placed on Muller Hinton agar plates (Hi Media, India) spread with ca. 10^5^ cells of actively growing test culture and incubated at 37°C. The inhibition zones measured after 24 h incubation and were interpreted according to the manufacturer’s interpretation table. MICs of antibiotics were determined by agar dilution method as described previously (Dhakephalkar and Chopade, 1994). Concentrations of each antibiotic used ranged from 1 μg/ml to 1,024 μg/ml. MIC was interpreted as the lowest concentration of the antibiotic inhibiting bacterial growth.

### Plasmid Isolation

Plasmid isolation was carried out by alkali lysis method as well as boiling method described by Sambrook et al. (1989). DNA was detected by horizontal agarose gel (0.7%) electrophoresis using Tris-acetate-EDTA buffer (pH 8.0).

### Curing of Antibiotic Resistance

The plasmid curing was performed as described earlier by Deshpande et al. (2001). In brief, microbial culture was exposed to different concentrations of curing agent during cultivation in Luria broth at 37°C for 24 h. Subsequently, the culture was serially diluted and plated on Luria agar to obtain isolated colonies. A total of 100 colonies was replica plated on Luria agar with and without antibiotic. Cured derivatives were scored by their failure to grow in presence of antibiotics. Efficiency of curing was calculated as number of colonies showing reversal of antibiotic resistance per 100 colonies tested. Agarose gel electrophoresis was performed to confirm the removal of plasmid DNA from the plasmid cured strain. Effect of pH (range 5.5–8.0), lawsone concentrations (range 64–512 μg/ml) and inoculum densities (range 10^4^–10^7^cells/ml) on curing efficiency of lawsone was studied in *E. coli* (pRK2013) and *A. baumannii* (pUPI281). All the experiments were performed at least in duplicate.

### Effect of Purified Active Principle (Lawsone) on Transformation of Plasmid

*E. coli* HB101was used as host for the transformation experiments. Calcium chloride method (Sambrook et al., 1989) was used for the preparation of competent cells. Transformation experiments were performed by “heat shock method” as described previously (Sambrook et al., 1989) using plasmid pRK2013 and competent cells of *E. coli* HB101 as recipient. Transformation efficiency was expressed as number of transformants obtained per μg of plasmid DNA. Purified active principle, at specified concentrations, was added to competent cells along with transforming DNA to evaluate its effect on plasmid transformation.

### Effect of Purified Active Principle (Lawsone) on Plasmid Transfer by Conjugation

Conjugation experiments were performed by membrane filter mating method as described by Towner and Chopade (1987). Effect of lawsone on plasmid transfer was studied by incubating both donor *E. coli* (pRK2013) and recipient *E. coli* HB101 (adsorbed on membrane filter) on nutrient agar containing varying concentrations of curing agent. Difference in the percentage efficiency of conjugation in the presence or absence of curing agent was calculated to evaluate effect of lawsone on transfer of plasmid by conjugation.

### Synergistic Action of Lawsone With the Antibiotic: Combination Studies

Synergism between lawsone and antibiotics such as streptomycin and kanamycin was tested against *A. baumannii* (pUPI281) and *E. coli* (pRK2013), respectively. Concentration of lawsone as well as the antibiotic tested was in the range 0–1,000 μg/ml. Actively growing culture was inoculated (10^4^ cells/ml) in Luria broth supplemented with varying concentrations of lawsone and streptomycin or kanamycin and incubated at 37°C for 18 h. Culture growth was monitored on spectrophotometer at 600 nm. Interactions of curing agents with antibiotics were assessed by a modified Chequerboard agar dilution method (Charles et al., 1997). Synergistic or otherwise effect of curing agent and antibiotic in combination was evaluated by determining the fractional inhibitory concentration index (minimum FIC index). The FIC was calculated for each combination using the following formula: **FIC A + FIC B = FICI** Where, FICA = MIC of drug A in combination/MIC of drug A alone, and FICB = MIC of drug B in combination/MIC of drug B alone. The FICI was interpreted as follows: synergy = FICI < 0.5; no interaction = FICI > 0.5–4; antagonism = FICI > 4 (Dorsthorst et al., 2002).

### Toxicity Testing of Lawsone

Actively growing BHK 21 (baby hamster kidney fibroblast cell line) and AV3 (human amniotic epithelial cell line) cells were obtained from the NCCS repository (Pune, India). These cells were subcultured using TPVG solution (2% trypsin, 0.1% EDTA, and 0.1% glucose in PBS) and seeded on to a 6-well tissue culture plate (Falcon, BD Biosciences, United States) at the concentration of 3 × 10^5^ cells per well with Eagle’s minimal essential medium (EMEM) supplemented with 10% fetal calf serum (FCS). Plates were incubated for 24 h for the monolayer formation. The monolayers of both, BHK 21 and AV3 were then exposed to different concentrations of lawsone. Plates were observed under inverted microscope (Olympus IX70) for the cell morphology and floating cell population after 24 h of incubation at 37°C in 5% CO_2_ environment. Control cells were grown in absence of lawsone.

### MTT Assay for Examination of the Viability of Cells

BHK21 and AV3 cells were seeded in a 96-well tissue culture plate (Falcon, BD Biosciences, United States) and incubated overnight. The cells were then exposed to varying concentrations of lawsone for 24 h. Control cells were not exposed to the lawsone treatment. MTT assay was performed to determine the toxicity of the plasmid curing agent to the cells upon exposure (Bahuguna et al., 2017) In this assay yellow colored 3-(4,5-dimethylazol-2-yl)-2,5-diphenyl-tetrazolium bromide (MTT) is enzymatically converted into purple formazan in the exposed cells. The intensity of purple formazan was measured at 570 nm. using Spectra Max 250 UV-vis microplate reader (Molecular Devices, Sunnyvale, CA, United States). Experiment was performed in duplicate and the average for all wells was shown as cell-viability percentage related to unexposed control cells.

## Results

### Purification and Identification of Plasmid Curing Agent From *P. zeylanica* Roots

**FIGURE 1:**
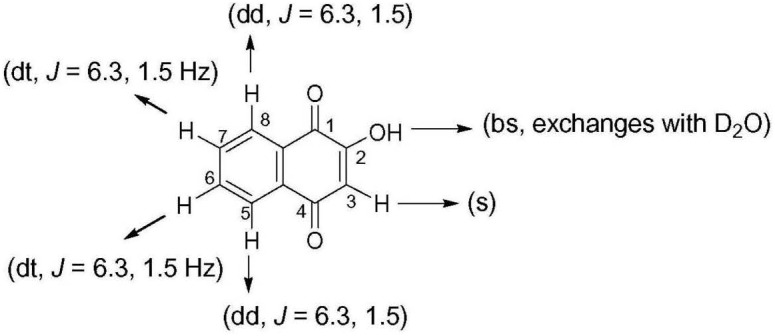
Chemical structure of lawsone demonstrating antimicrobial and plasmid curing activity.

Crude ethanol extract of roots of *P. zeylanica* demonstrated significant antimicrobial as well as plasmid curing activity (Patwardhan, 2007). An active principle responsible for the plasmid curing activity of the ethanol extract of *P. zeylanica* was purified by bioassay guided fractionation. Molecular formula of the purified compound, isolated as yellow crystals, (melting point, 192°C) was established as C_10_H_6_O_3_ (Figure 1) based on GCMS spectral data (molecular ion peak at *m/z* = 174). Further structural characterization was made by UV, FTIR, ^1^H NMR and ^13^C NMR spectroscopic methods. UV λ_max_ at 273 nm for the same compound clearly indicated conjugation in the compound. In the IR spectra, the peak observed at 1,587 cm^-1^ suggested the presence of C=C. The peak at 1,641 cm^-1^ indicated the presence of α, β-unsaturated carbonyl group. The broad band at 3,056–3,350 cm^-1^ was suggestive of hydroxyl group. In the ^1^H-NMR spectrum, the singlet at 6.36 δ was assigned to H-3. The broad singlet 7.33 was due to hydroxyl group which was found to be exchangeable with D_2_O. The signal at δ 7.71 appeared as doublet of triplet with *J* = 6.3 and 1.5 Hz was assigned to either H6 or H7. Similarly, appearance of doublet of triplet at δ 7.79 with *J* = 6.3 and 1.5 Hz was assigned to either H6 or H7. The signal at δ 8.11, corresponding to two protons, appeared as doublet of doublet with *J* = 6.3 and 1.5 Hz was assigned to H5 and H8. The compound was identified as lawsone based on spectral data (Figure 1 and Table 3). This data was found to be in good agreement with the spectral and analytical data of lawsone (Sigma, United States) thus confirming our assignment. To the best of our knowledge, this is the first report of lawsone extracted from roots of *Plumbago zeylanica*.

**Table 3:** ^1^H NMR and ^13^ C NMR data of lawsone.

Position	^1^H NMR (δ)	Position	^13^ C NMR (δ)
H-3	6.19 (S)	C-1	184.9
H-5	8.19 (dd, *J* = 6.3 and 1.4 Hz)	C-2	156.3
H-6	7.72 (dt, *J* = 6.3 and 1.4 Hz)	C-3	110.7
H-7	7.81 (dt, *J* = 6.3 and 1.4 Hz)	C-4	181.9
H-8	8.19 (dd, *J* = 6.3 and 1.4 Hz)	C-4a	132.9
		C-5	133.13
		C-6	126.7
		C-7	126.4
		C-8	135.3
		C-8a	129.4

### Curing of Antibiotic Resistance by Lawsone

**FIGURE 2:**
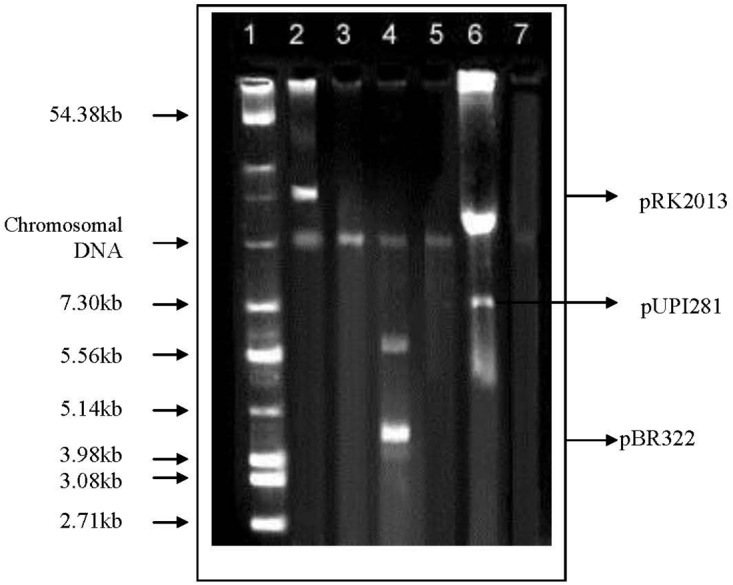
Agarose gel electrophoresis of plasmid DNA in standard strains as well as clinical isolates harboring R-plasmids and their cured derivatives. Lane 1, Reference plasmids from *E. coli* MTCC 131; Lane 2, *E. coli* MTCC398 (pRK2013); Lane 3, Cured derivative of *E. coli* MTCC398; Lane 4, *E. coli* K12 (pBR322); Lane 5, Cured derivative of *E. coli* K12; Lane 6, *Ac. baumannii* A24 (pUPI281); Lane 7, Cured derivative of *Ac. baumannii* A24.

Lawsone inhibited the growth of *A. baumannii*, *P. aeruginosa*, *S.* Typhi and *E. coli*, in disk diffusion assay (200–800 μg/disk) (Table 1). Plasmid curing activity of lawsone against R-plasmids harbored in reference as well as clinical isolates is illustrated in Table 2 and Supplementary Figures S1, S2. Lawsone cured plasmids pBR322 and pRK2013 at 11% and 20% efficiencies, respectively. Lawsone also cured plasmid R136 in *S.* Typhi at 4.2% curing efficiency. However, it was not able to cure plasmid RP4 in *E. coli*. Lawsone eliminated plasmids pUPI281 and pUPI282 in *A. baumannii* with 8.5% and 13.5% efficiency, respectively. Curing efficiencies of lawsone observed against plasmids in reference strains as well as clinical isolates of *A. baumannii* were comparable to those observed for conventional curing agents such as DNA-intercalating dyes, acridine orange and ethidium bromide (Table 4). Plasmid curing efficiency of lawsone was determined against plasmid pBR322, pRK2013, and pUPI281 at different cell densities of host harboring these plasmids. It was observed that curing efficiencies were higher at lower cell densities (10^4^ cells/ml) than at higher cell densities (10^5^ cells/ml or 10^6^ cells/ml) (Table 5). Similarly, increasing concentrations of lawsone (64–512 μg/ml) resulted in higher curing efficiencies when initial inoculum density was maintained constant (Table 6). Lawsone could not cure either pUPI281 or pRK2013 at 64 μg/ml or lower concentrations. Plasmid curing activity of lawsone was studied at different pH ranging from 6.0 to 8.0 with plasmids pRK2013 and pUPI281. It was observed that lawsone cured pRK2013 and pUPI281 with curing efficiency of 27% and 17%, respectively at pH 6.0 and 8.0. There exists a possibility of reversal of antibiotic resistance if and when a bacterial culture is grown in absence of antibiotics. Hence, in the present study bacterial culture was grown in absence of antibiotics as well as plasmid curing agent (solvent control). Such culture was plated to obtain isolated colonies in absence of antibiotics. The isolated colonies were replicated on agar media with or without antibiotic to determine the frequency of spontaneous loss of plasmid or reversal of antibiotic resistance. None of the 100 colonies tested were spontaneously cured derivative of plasmid bearing host. However, in this technique only 100 colonies were tested by replica plating. Hence, in another modification, cultures were grown in absence of antibiotics or plasmid curing agents for several generations and then plated on agar media with and without antibiotics. The frequency of spontaneous reversal of antibiotic resistance was found to be less than one in 10^8.^ This observation was consistent with earlier observation (Inoue, 1997). In comparison, curing efficiencies reported in this study were extremely high (>10^4^ times). Both these observations (reversal of antibiotic resistance or plasmid curing observed only upon exposure to plasmid curing agents and not spontaneously) clearly indicated that reversal of antibiotic resistance as a consequence of plasmid curing was not a spontaneous phenomenon but was caused by the exposure of plasmid harboring strains to plasmid curing agent, i.e., lawsone in the present study. In the present investigation, loss of plasmid in the cured derivatives was confirmed by agarose gel electrophoresis analysis (Figure 2). Loss of plasmid and not mutation was thus, confirmed as a cause of reversal of antibiotic resistance in the cured derivative. It may be noted here that even mutation could have caused reversal of antibiotic resistance. However, mutagenic activity of the plasmid curing agents is undesired for potential clinical application. Lawsone was able to cure plasmid and inhibit the transfer of plasmid by conjugation as well as by transformation which is significant in containing the spread of antibiotic resistance. Controls did not show loss of plasmids at various pH values, indicating that pH alone did not have any effect on the plasmid curing.

**Table 4:** Curing of R plasmids with conventional curing agents.

		Purified lawsone		Ethidium bromide		Acridine orange	
Plasmid	Antibiotic resistance markers	Conc. (μg/ml)	Curing efficiency	Conc. (μg/ml)	Curing efficiency	Conc. (μg/ml)	Curing efficiency
			**(%)**		**(%)**		**(%)**
RP4	Ap^r^, Km^r^, Tc^r^	256	_	128	13 ± 1.4	64	ND
pBR 322	Ap^r^, Tc^r^	512	11 ± 1.4	128	21.5 ± 2.1	128	12.5 ± 2.1
pRK 2013	Ap^r^, Km^r^	512	20 ± 1.4	256	59.5 ± 3.5	128	ND
RIP64	Gm^r^, Cm^r^	256	_	128	16	256	ND
R136	Tc^r^	512	4.2 ± 0.3	256	13.5 ± 0.7	256	ND
pUP110	Km^r^, Nm^r^	512	_	256	_	128	ND
pUPI 280	Ap^r^, Gm^r^, Km^r^, Cm^r^, Am^r^	800	_	512	18 ± 2.8	512	6.5 ± 0.7
pUPI 281	St^r^, Ap^r^, Gm^r^, Ak^r^	512	13.5 ± 1.4	256	15 ± 1.4	256	17.25 ± 1.06
pUPI 282	St^r^, Ap^r^, Gm^r^, Ak^r^, Lf ^r^	256	8.5 ± 0.7	256	10.25 ± 1.0	256	_

*Three hundred clones were tested for each plasmid in the curing experiments. ND, none of the 300 colonies tested showed curing of plasmid*.

**Table 5:** Effect of cell density on plasmid curing efficiency of lawsone.

Plasmid	Curing efficiency (%) at			
	10^4^ (cells/ml)	10^5^ (cells/ml)	10^6^ (cells/ml)	10^7^ (cells/ml)
*E. coli* (pBR 322)	31.3 ± 1.8	16.5 ± 2.1	2 ± 0	ND
*E. coli* (pRK 2013)	20 ± 1.4	9.3 ± 2.5	4.5 ± 0.7	9 ± 0
*A. baumannii* (pUPI 281)	12.5 ± 2.1	15.5 ± 2.1	15 ± 0	5.8 ± 1.0

*Above desired number of cells were incubated in Luria broth in the presence of 512 μg of lawsone/ml at 37°C for 24 h. Number of clones tested – 300. ND, none of the 300 colonies tested showed curing of plasmid*.

**Table 6:** Effect of concentration of lawsone on curing efficiency.

Plasmid	Curing efficiency (%) at			
	64 (μg/ml)	128 (μg/ml)	256 (μg/ml)	512 (μg/ml)
*E. coli* (pBR 322)	ND	ND	13 ± 1.4	31.74 ± 1.06
*E. coli* (pRK 2013)	ND	ND	20 ± 1.4	22 ± 1.4
*A. baumannii* (pUPI 281)	ND	7.5 ± 2.1	18 ± 1.4	14.3 ± 0.4

*Number of clones tested – 300. 10^4^ cells/ml were incubated in the presence of above described concentrations of lawsone at 37°C for 24 h. ND, none of the 300 colonies tested showed curing of plasmid*.

### Effect of Lawsone on Plasmid Transfer by Conjugation and Transformation

Effect of lawsone on plasmid transfer by conjugation or transformation was investigated with plasmid pRK2013. More than 90% decrease in the frequency of plasmid transfer was observed when conjugation was performed in the presence of lawsone (12.5 μg/ml) by membrane mating method. Plasmid pRK2013 was transferred from *A. baumannii* to *E. coli* with conjugation frequencies of 2.1 × 10^-5^ and 2 × 10^-4^ in the presence and absence of lawsone, respectively (Table 7). The similar inhibition was observed when the conjugation was performed by broth mating (8.8 × 10^-6^ and below detection limit). Similarly, lawsone inhibited transfer of plasmid by transformation. Frequency of transformation of *E. coli* HB101 with pRK2013 was observed to be 1.1 × 10^4^ transformants/μg plasmid DNA. However, 63% decrease in the transformation efficiency was observed in the presence of lawsone.

**Table 7:** Effect of lawsone on R plasmid transfer from ampicillin resistant *E. coli* to streptomycin resistant *E. coli* by conjugation.

Conjugation	Frequency of conjugation	
	In absence of curing agent	In presence of curing agent
With membrane filter	2 × 10^-4^	2.1 × 10^-5^
Without membrane filter	8.8 × 10^-6^	Below the detection limit

*10^8^ cells of donor *E. coli* (pRK2013) and recipient *E. coli* (HB101) were mixed and incubated in absence or presence of 12.5 μg of lawsone/ml at 37°C for 24 h. The growth was serially diluted and plated out to score number of transconjugants formed. The data represents average values of two parallel experiments*.

### Synergistic Action of Lawsone With the Antibiotic: Combination Studies

Synergy between lawsone and antibiotics was investigated against *A. baumannii* (pUPI281) and *E. coli* (pRK2013). The MICs were determined for the curing agent alone and in combination with the different antibiotics by checkerboard assay method. *A. baumannii* (pUPI281) and *E. coli* (pRK2013) were resistant to streptomycin and kanamycin, respectively (MIC > 1,000 μg/ml). Growth of *A. baumannii* (pUPI281) was inhibited when lawsone and streptomycin were added together at 250 μg/ml concentrations each. Similarly, growth of *E. coli* (pRK2013) was inhibited when lawsone and kanamycin were added together at 100 μg/ml and 250 μg/ml concentrations, respectively. The Fractional Inhibition Concentration (FIC) indices were calculated (Table 8). These results clearly indicated that FICI was synergistic (<0.5) for lawsone in combination with antibiotic when used against *A. baumannii.* Additive action of lawsone with antibiotic was observed against *E. coli*. These results suggested that the plasmid curing activity of naphthoquinone directly or indirectly rendered the clinical isolates susceptible to the inhibitory action of antibiotics at significantly lower concentration, and hence, were considered encouraging.

**Table 8:** FIC index of individual curing agents and in combination with antibiotics.

Test organism	FIC_c_^∗^	FIC_a_^@^	FIC_i_^#^	Interpretation
*A. baumannii*	0.25	0.133	0.383	Synergistic
*E. coli*	0.4	0.25	0.65	Additive

*^∗^Fractional Inhibition Concentration of curing agent (lawsone). ^@^Fractional Inhibition Concentration of antibiotics. ^#^Fractional Inhibition Concentration index*.

### Toxicity Testing and Viability Assay (MTT) of Lawsone

**FIGURE 3:**
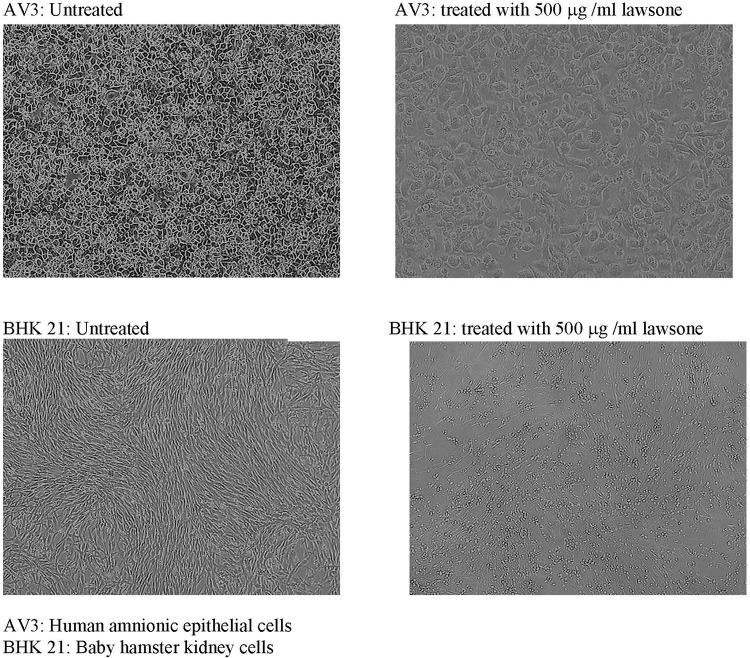
Toxicity testing of lawsone on AV3 and BHK 21 cell lines. AV3, human amnionic epithelial cells; BHK 21, baby hamster kidney cells.

Toxicity testing of lawsone revealed that BHK 21 cells which are fibroblastic in nature are more affected with lawsone than AV3 cells which are epithelial indicating its selective toxicity to fibroblast only. In MTT assay BHK 21 cells showed more drop in % viability with increased concentration of lawsone compared to AV3 Cells (Table 9 and Figure 3).

**Table 9:** Toxicity testing with MTT assay.

Conc. (μg/ml)	Cell line used			
	BHK 21		AV3	
	
	Average absorbance	% Viable cells	Average absorbance	% Viable cells
100	0.7715	84.8268	1.1035	98.55
200	0.6625	72.8422	1.1395	97.27
300	0.6650	73.1170	1.064	90.82
400	0.5995	65.9153	0.99	84.50
500	0.6275	68.9939	0.897	76.57
Control blank	0.9095	100	1.1715	100

*BHK 21, baby hamster kidney fibroblast cells; AV3, human amnionic epithelial cells. Formula used: % viable cells = (average of test OD/average of control OD) × 100*.

## Discussion

The bioassay guided fractionation of the organic extracts of *P. zeylanica* roots resulted in identification of lawsone as an active principle exhibiting the plasmid curing activity. Lawsone has been reported to be a major constituent of *Lawsonia inermis.* However, to the best of our knowledge, this is the first report of lawsone extracted from roots of *Plumbago zeylanica*. Antimicrobial properties of lawsone have been documented in the literature (Ahmed and Beg, 2001).

Treatment of chronic infections caused by multidrug resistant bacteria is a serious challenge for the physicians. In most of the cases the resistance to multiple antibiotics is mediated by the plasmids. Curing of plasmid-mediated antibiotic resistance in pathogenic strains of bacteria is of great practical importance both in treatment of bacterial infection and in microbial genetics. In the present investigation, curing of plasmids effected by lawsone was confirmed on the basis of reversal of antibiotic resistance phenotype evident from the significant reduction in the MIC of antibiotics; as well as elimination of plasmids in the cured derivatives. Agarose gel electrophoresis revealed presence of plasmid DNA in wild host. However, gel electrophoresis of DNA from cured derivatives revealed loss of the corresponding plasmid band confirming plasmid curing. Plasmid curing was not observed in host exposed to DMSO, a solvent control used for lawsone. Spontaneous loss of plasmid DNA has been reported at low frequency of less than 1 in 10^9^ cells^.^ (Inoue, 1997). In comparison curing efficiencies observed in the present study were significantly higher (>10^4^ times), thus attributing reversal of antibiotic resistance to the exposure of cells to the curing agent (lawsone). Cured strains were tested for the loss of plasmids by agarose gel electrophoresis to confirm that reversal of antibiotic resistance was due to elimination of plasmid carrying genes encoding resistance to antibiotics and not due to mutations. Physical loss of plasmid as evidenced by agarose gel electrophoresis of plasmid DNA preparation of cured strains (Figure 2) indicated that genes encoding antibiotic resistance were located on plasmid and that plasmid loss resulted in subsequent loss of antibiotic resistance.

Ability of lawsone to cure plasmid encoded antibiotic resistance in *Acinetobacter* strains is particularly significant since *Acinetobacter* strains are known to act as a reservoir of natural or acquired antibiotic resistance genes in the nosocomial environment facilitating in the spread of antibiotic resistance genes to more pathogenic bacteria (Towner, 1997; Deshpande et al., 2001; Cisneros and Rodriguez-Bano, 2005). Bacteria have tendency to develop resistance against any antimicrobial agent used against them. Concentrations of curing agents used in the plasmid curing experiments were significantly lower than inhibitory concentrations. Hence, it is proposed that chances of bacteria developing any mechanism to inactivate plasmid curing agents or their activity are extremely low. It must be remembered here that plasmids are after all dispensable elements in a bacterial cell.

Plasmids are the extrachromosomal elements that are responsible for development and spread of antibiotic resistance in bacteria. Their role assumes even more significance in the nosocomial environment as plasmid encoded resistance to multiple antibiotics can be transferred from one host to another by inter species transfer modes such as conjugation and/or transformation. Such acquired resistance can make otherwise sensitive pathogens resistant to multiple antibiotics. Thus, making the treatment of infections more difficult and in some cases almost impossible. Ability of lawsone to interfere with interspecies plasmid transfer by conjugation and transformation and thus contain the spread of multi-resistance to antibiotics was investigated in the present study. It was observed that Lawsone not only cured plasmids but also inhibited the transfer of plasmid by both conjugation as well as by transformation. Membrane filter mating technique and broth mating are the two different techniques used to investigate plasmid transfer by conjugation in bacteria. Lawsone was able to inhibit the plasmid transfer by conjugation in both techniques. *Acinetobacter* has been reported as a reservoir of antibiotic resistance genes in nosocomial environment and is frequently reported to be involved in the transfer of multi-resistance to antibiotics. *E. coli* has been the most extensively investigated organism for the inter species gene transfer. Hence, in the present study both organisms were included to investigate the effect of lawsone on inter species plasmid transfer. Ability of lawsone to inhibit plasmid transfer among these organisms may thus assume special significance in nosocomial environment. Ability of lawsone to inhibit plasmid transfer by transformation or conjugation was reported for the first time in the present investigation.

FIC clearly indicated that combination of curing agent and antibiotic was synergistic against *A. baumannii* and additive against *E. coli*. This observation was considered encouraging as it suggested that the plasmid curing activity of naphthoquinone rendered the clinical isolates susceptible to the inhibitory action of antibiotics at significantly lower concentration. Efficacy of extended antibiotic therapy in several microbial infections may be improved with the combination of plasmid curing agent lawsone, as a synergistic drug. Such synergistic combination of antibiotic and lawsone, may serve as a prospective device in selection of appropriate drug therapy that is likely to contribute to the ongoing crusade against microbial drug-resistance.

Plant derived compounds have been previously reported as plasmid curing agents. Such compounds included 8-epidiosbulbin E acetate isolated from the bulbs of *Dioscorea bulbifera* (Shriram et al., 2008); 1’-acetoxychavicol acetate from *Alpinia galanga* (L.) Swartz, (Latha et al., 2009); Plumbagin (5-hydroxy-2-methyl-1,4- naphthoquinone) derived from the root of the tropical/subtropical Plumbago species (Patwardhan et al., 2015), etc. These plasmids curing agents have been proven to be effective at curing plasmids *in vitro*. Unsaturated fatty acids have been shown to be effective conjugation inhibitors in laboratory studies on a variety of plasmids (Lopatkin et al., 2017). Furthermore, they are associated with reduced toxicity on tissue culture cells. Lawsone, a plant derived compound reported in the present investigation has shown dual ability to cure plasmid and also inhibit interspecies plasmid transfer. However, more research is needed to confirm *in vivo* efficacy and to determine potential toxicity of these compounds if administered *in vivo*.

Sauriasari et al. (2007) reported that lawsone was not mutagenic to bacterial strains. However dose dependent cytotoxicity of lawsone was reported in the same study. Cytotoxicity of lawsone was reported to be significantly lower than that of other naphthoquinone derivatives including cisplatin, a widely used anticancer drug (Oliveira et al., 2017). Cytotoxicity of naphthoquinones such as lawsone could be attributed to generation of reactive oxygen species, disruption of mitochondrial functions, inhibition of thymidine incorporation into DNA and DNA intercalation (Aithal et al., 2009; Bonifazi et al., 2010; Klaus et al., 2010; Babula et al., 2012). A series of tests in the published literature have identified lawsone as non-genotoxic agent. Such tests included Ames test, V79 hprt test, Syrian hamster embryo cell transformation assay, bone marrow micronucleus tests in CD1 mice, and bone marrow chromosome aberration test in mice and hamsters (Kirkland and Marzin, 2003). An additional genetic toxicity program performed to clarify *in vivo* genotoxic potential of lawsone revealed that lawsone was devoid of clastogenic potential *in vivo* (Marzin and Kirkland, 2004). Thus, the review of literature illustrating evaluation of genotoxicity of lawsone in a series of *in vivo* and *in vitro* tests has revealed that lawsone is not genotoxic when administered orally up to a dose of 300 mg/kg body weight. Ability of lawsone to intercalate with double stranded DNA has been reported in the published literature (Kirkland and Marzin, 2003). Also, the ability of lawsone to alter the cell membrane by generating free radicle stress has been reported earlier (Sauriasari et al., 2007). However, the plasmid curing activity reported in the present study was observed at concentration significantly lower than the inhibitory concentrations. Elucidation of exact mechanism by which lawsone effected plasmid curing in bacterial scale is not known at present and requires further extensive investigation.

Thus, in conclusion, this investigation has revealed a compound with an ability to eliminate antibiotic resistance and cure plasmids from pathogenic strains that are resistant to multiple antibiotics without any ill effect on mammalian cells at lower concentrations. The results obtained provide endorsement for investigating the potential value of lawsone in combating clinical drug resistance. The synergistic effect of lawsone with the antibiotic exhibits its tremendous potential in modern day therapeutics. The non-toxic, non-mutagenic, plasmid curing and plasmid transfer inhibiting role of lawsone demands further investigation to make it a potential drug of choice in the treatment of antibiotic resistant bacterial strains; thus, demonstrating a new dimension in antibiotic therapy.

## Author Contributions

RP, PD, and BC designed and executed the experiments. RP performed the experiments. RP, PD, BC, DD, and RB interpreted the results and analyzed the data. RP, PD, DD, and RB contributed to the writing of the manuscript. RP and PD revised the manuscript.

## Conflict of Interest Statement

The authors declare that the research was conducted in the absence of any commercial or financial relationships that could be construed as a potential conflict of interest.
