# Supplementary material for: Purification and Characterization of an Active Principle, Lawsone, Responsible for the Plasmid Curing Activity of Plumbago zeylanica Root Extracts
Source: Front Microbiol. 2018 Nov 8;9:2618. doi: 10.3389/fmicb.2018.02618 (PMC6236066; doi:10.3389/fmicb.2018.02618)
Supplement: Supplementary file 1 [file Data_Sheet_1.PDF]

Supplementary Material:

Results:

1. Plasmid curing experiments support with images of changes in colony number (supplementary)

Fig1. Plasmid curing in *E. coli* (pRK2013)

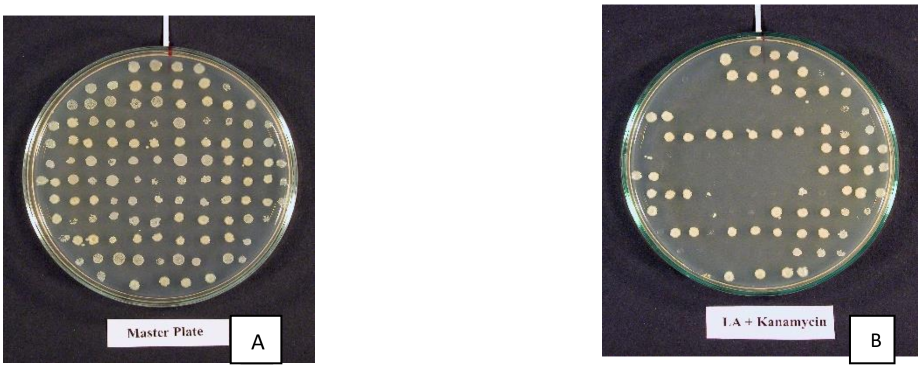

A: Master plate with growth of *E. coli* (pRK2013) clones on complete medium Luria agar (LA); B: Replica plate (LA+ Kanamycin) with presence (non cured) and absence (cured

Fig.2. Plasmid curing in *A. baumannii* (pUPI281) A24 with *P. zeylanica* ethanol root extract

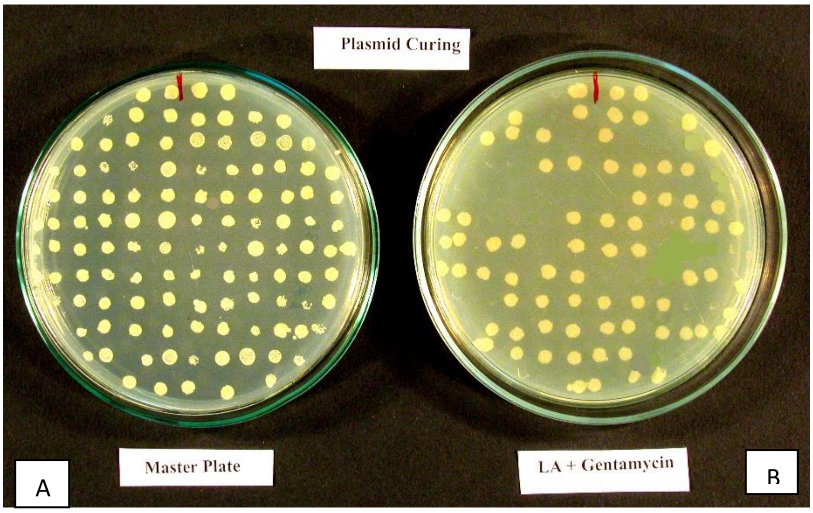

A: Master plate-Luria agar; B: Replica plate
